# Implementation Research: An Efficient and Effective Tool to Accelerate Universal Health Coverage

**DOI:** 10.15171/ijhpm.2019.125

**Published:** 2019-12-09

**Authors:** Agnes Binagwaho, Miriam F. Frisch, Kelechi Udoh, Laura Drown, Jovial Thomas Ntawukuriryayo, Dieudonné Nkurunziza, Kateri B. Donahoe, Lisa R. Hirschhorn

**Affiliations:** ^1^University of Global Health Equity, Kigali, Rwanda.; ^2^Department of Medical Social Sciences, Feinberg School of Medicine, Northwestern University, Chicago, IL, USA.

**Keywords:** Implementation Research, Universal Health Coverage, Evidence-Based Decision-Making, Embedded Research

## Abstract

Success in the implementation of evidence-based interventions (EBIs) in different settings has had variable success. Implementation research offers the approach needed to understand the variability of health outcomes from implementation strategies in different settings and why interventions were successful in some countries and failed in others. When mastered and embedded into a policy and implementation framework, the application of implementation research by countries can provide policy-makers and implementers with the knowledge necessary to work towards universal health coverage (UHC) with the effectiveness, efficiency, sustainability, and fidelity needed to achieve sustainable positive health outcomes for all. To achieve this goal however, work is needed by the communities of research producers and consumers to create more clarity on implementation research methodologies and to build capacity to apply them as a critical tool for countries on their path to achieving UHC.

Scientific innovations in preventive, curative, palliative, and rehabilitative medicine have dramatically improved access to more effective care and treatment across the world.^1,2^ However, implementation approaches for interventions that generated positive health outcomes in one place have not always been successful elsewhere, even when conditions are similar. This variability demonstrates the need for better evidence for countries to identify the appropriate implementation strategies to ensure success and the contextual factors which inform where and how adaptation is needed.^3,4^ To understand why the population level success of an evidence-based intervention (EBI) such as vaccination is not uniform when implemented in real world settings, a new type of science, implementation science (Table), has emerged in prominence over recent years.

**Table:** Implementation Science Terms and Definitions

**Term**	**Definition**
Implementation science	“The study of methods to promote the adoption and integration of evidence-based practices, policies, research findings and evidence into healthcare policy and practice.”^5^
Implementation research	“The scientific study of the use of strategies to adopt and integrate evidence-based health interventions into clinical and community settings to improve individual outcomes and benefit population health.”^6^
Implementation strategies	The approaches used to get the interventions such as vaccinations implemented and sustained, representing the decisions and work done to move evidence into general practice.^7^
Context	“The set of circumstances or unique factors that surround a particular implementation effort.”^8^
EBIs	Interventions shown to have efficacy and effectiveness in more controlled settings.^9^

Abbreviation: EBIs, evidence-based interventions.

Done well, implementation research can facilitate the scale-up and sustainment of EBIs and policies.^10^ These methods also recognize the role of contextual factors, their importance in the successes and failures of implementation strategies, and where adaptation is needed in different settings. ^8,11^ These factors include resources and the design of health systems, stakeholders within healthcare delivery systems, including healthcare professionals and other implementers, and the community being served, including their values, attitudes and resources. Organizational behavior, at the national and local levels, is also an important factor because it affects decision-making and action from planning stages through implementation and sustainability of the evidence-based practices.^8,11^ Through implementation research, policy-makers, healthcare professionals, and researchers now have a set of methods to study when, how, and why implementation strategies, policies, and laws designed to move evidence from research into practice can be successfully tested, routinely adapted, and integrated into policy and practice to achieve desired population health outcomes.

In the health sector, adopting and embedding implementation research into health care policy and practice will accelerate progress to the global goals of universal health coverage (UHC), which is defined as access to affordable quality healthcare, essential for reaching the goals of improving healthcare for all.^12^ Implementation research can generate better knowledge needed to answer the key questions that face policy-makers, politicians, implementers, and the community on how to accelerate and strengthen implementation, effectiveness, and sustainability of interventions known to improve individual and population health and reach. In particular, the knowledge on when and how to choose and adapt implementation strategies to local contexts, within and across countries, is important and often missing. This knowledge gained can be applied to both lower- and higher-income settings, though our analysis is focused on lower-income countries as it is our area of expertise. Some critical areas where implementation research can produce the needed knowledge include:

1. **How implementation strategies** are optimally designed and put into practice to increase access to quality care and save lives. This work can be done through implementation research frameworks such as Exploration, Preparation, Implementation and Sustainment or determinant frameworks such as Consolidated Framework for Implementation Research.^13,14^ These frameworks can guide how to design and put into action an overall implementation plan, how to adopt or adapt effective implementation strategies, including when and how to incorporate systems and societal context into implementation decisions. Applied systematically, the use of implementation research will promote a learning environment when strengthening health systems, and better equip decision-makers and implementers to increase quality service coverage of current and future EBIs, as well as to respond to unpredictable events which have become increasingly frequent.^15^
2. **Which contextual factors** are most important to consider while designing the implementation of interventions. These factors include those which should influence the choice and adaption of implementation strategies, factors which can be leveraged to increase success, and other factors which need to be addressed as threats to achieving the goals targeted by the intervention. For example, implementation of efforts to expand family planning would differ based on cultural norms, community trust in the health care system, health care worker availability, and access to health care facilities.^16,17^
3. Why and how countries decide to **invest in scaling up** of implementation strategies from initial testing of interventions to achieve greater access to quality care nationally. There is important knowledge gained by studying how and why the initial testing worked and how to expand and adapt the implementation strategies as needed. Additionally, knowledge on how to embed measurement of key implementation outcomes (including acceptability, appropriateness, feasibility, fidelity, sustainability, and cost) is important to increase planning capacities, understand what data are needed in different contexts, and accelerate country coverage.^18^
4. **How to accelerate new EBI implementation** (within a country or elsewhere) through implementation research in order to produce knowledge on effective implementation strategies and key contextual factors which can then be applied to new EBIs. This use of generalizable knowledge can decrease the time between the development of new scientific innovations and their full and effective implementation, allowing countries to save more lives more quickly. For example, in Rwanda, the human papilloma virus vaccine was implemented based on knowledge gained from other vaccine implementation, with adaption such as for the different age group.^19^
5. **How to ensure sustainability** by understanding what strategies were used by countries who have sustained implementation of EBIs. These include strategies used during the planning and implementation phases, such as integrating them into their planning and budgeting and the regulatory processes, as well as leveraging in-country resources and other system changes needed to sustain improved health outcomes.

If applied effectively by countries working to reach the goals of UHC, implementation research can not only improve access to evidence-based healthcare services but make the implementation more efficient and these services more effective and create a system more ready to respond to new or emerging needs. However, if implementation research is to be useful for reaching UHC, much work remains. There are different schools of thought, processes, and terminology in implementation research, leading to confusion and lack of consensus among potential consumers and emerging researchers from countries in definitions and how to apply and communicate methods and results.^20^ There also remains a lack of consensus around how to best apply these methods and frameworks in different settings to generate the different types of evidence needed depending on the intervention under consideration or implementation gap being addressed.^5^ This ambiguity limits policy-makers’ and researchers’ ability to make decisions on what research and evidence is needed and the conclusions they can draw about research results, including which implementation approaches are replicable and which need adaptation for more effective scale.^20^

If we can achieve this clarity and the needed country capacity and ownership, when mastered and embedded into policy and implementation work, implementation research can produce the knowledge needed to determine how to reach UHC with maximum efficiency, equity, fidelity, and sustainability. Building the capacity of in-country researchers and consumers of the new knowledge, including implementers and policy-makers, is an important step needed to ensure the results produced are relevant to the setting and accelerate the uptake through improved ownership and engagement.^21^

## Ethical issues

Not applicable.

## Competing interests

Authors declare that they have no competing interests.

## Authors’ contributions

AB: Conception and design; LRH: Assisted with conception and design, critical revision of the manuscript for important intellectual content; MFF, LD, KBD, JTN, DN, KU, DN: Critical revision of the manuscript for important intellectual content; DN: Administrative support.

## Authors’ affiliations

^1^University of Global Health Equity, Kigali, Rwanda. ^2^Department of Medical Social Sciences, Feinberg School of Medicine, Northwestern University, Chicago, IL, USA.
